# Influence of Anticoagulants on the Dissociation of Cardiac Troponin Complex in Blood Samples

**DOI:** 10.3390/ijms25168919

**Published:** 2024-08-16

**Authors:** Natalia S. Riabkova, Alexander E. Kogan, Ivan A. Katrukha, Alexandra V. Vylegzhanina, Agnessa P. Bogomolova, Amina K. Alieva, Dmitry V. Pevzner, Anastasia V. Bereznikova, Alexey G. Katrukha

**Affiliations:** 1 HyTest Ltd., Intelligate 1, 6th Floor, Joukahaisenkatu 6, 20520 Turku, Finland; natalia.riabkova@hytest.ru (N.S.R.); alexander.kogan@hytest.ru (A.E.K.); avylegzhanina@gmail.com (A.V.V.); agnessa.bogomolova@hytest.ru (A.P.B.); anastasia.bereznikova@hytest.ru (A.V.B.); alexey.katrukha@hytest.fi (A.G.K.); 2 Department of Biochemistry, Faculty of Biology, Moscow State University, Leninskie Gory 1, str. 12, 119234 Moscow, Russia; 3 National Medical Research Centre of Cardiology Named after Academician E.I. Chazov, Akademik Chazov str., 15A, 121552 Moscow, Russia; amina_alieva_1998@mail.ru (A.K.A.); pevsner@mail.ru (D.V.P.)

**Keywords:** cardiac troponin complex, proteolytic degradation, dissociation, myocardial infarction, anticoagulants, biomarkers

## Abstract

Immunodetection of cardiac isoforms of troponin I (cTnI) and troponin T (cTnT) in blood samples is widely used for the diagnosis of acute myocardial infarction. The cardiac troponin complex (ITC-complex), comprising cTnI, cTnT, and troponin C (TnC), makes up a large portion of troponins released into the bloodstream after the necrosis of cardiomyocytes. However, the stability of the ITC-complex has not been fully investigated. This study aimed to investigate the stability of the ITC-complex in blood samples. A native ITC-complex was incubated in buffer solutions, serum, and citrate, heparin, or EDTA plasma at various temperatures. Western blotting and gel filtration were performed, and troponins were detected using specific monoclonal antibodies. The ITC-complex dissociated at 37 °C in buffers with or without anticoagulants, in citrate, heparin, and EDTA plasmas, and in serum, into a binary cTnI-TnC complex (IC-complex) and free cTnT. In plasma containing heparin and EDTA, the IC-complex further dissociated into free TnC and cTnI. No dissociation was found at 4 °C or at room temperature (RT) in all matrices within 24 h except for EDTA plasma. After incubation at 37 °C in EDTA plasma and serum, dissociation was accompanied by proteolytic degradation of both cTnI and cTnT. The presence of anti-troponin autoantibodies in the sample impeded dissociation of the ITC-complex. The ITC-complex dissociates in vitro to form the IC-complex and free cTnT at 37 °C but is mostly stable at 4 °C or RT. Further dissociation of the IC-complex occurs at 37 °C in plasmas containing heparin and EDTA.

## 1. Introduction

The troponin complex (ITC-complex) comprises troponin I, troponin T and troponin C (TnC) [1,2] and plays a key role in the regulation of muscle contraction [3,4]. The binding of calcium and magnesium ions by TnC is crucial for complex formation and function [5,6]. Cardiac muscle contains the tissue-specific cardiac isoforms of troponin I (cTnI) [7] and troponin T (cTnT) [8], which can be used as biomarkers of myocardial damage [9,10]. Upon acute myocardial infarction (AMI), the ITC-complex is released into the bloodstream and is measured in blood samples via its cTnI or cTnT components [11,12,13]. Various fragments of cTnI and cTnT can also be detected in the bloodstream [14,15]. 

We demonstrated that the ITC-complex circulates in the blood in several forms: a ternary full-size ITC-complex, a ternary low-molecular-weight complex (LMW ITC-complex) in which cTnT is reduced to a C-terminal fragment, and a binary cTnI-TnC complex (IC-complex) [16]. Investigation of plasma samples taken from patients with AMI at different time points showed that at the early stage of the disease, the proportions of the full-size and LMW ITC-complexes were high, whereas the binary IC-complex became predominant in blood samples 20–30 h after AMI onset [16]. 

Whether all types of Tn-complexes form in the damaged myocardium and are then released into the bloodstream, or if the dissociation takes place in circulation is not known. The extent to which the ratio of different troponin complexes in patient circulation corresponds to the ratio detected in blood samples taken for analysis is also unclear. Modern immunodiagnostic systems utilise various types of blood samples for the measurement of troponins. However, the influence of the most common anticoagulants (sodium citrate, heparin, or EDTA) on the integrity of the ITC-complex remains unclear.

The aim of the present study was to investigate the influence of various anticoagulants on the stability of Tn-complexes in blood samples.

## 2. Results

### 2.1. Time Course of Immunochemical Activity of the ITC-Complex in Different Matrices

**Figure 1:**
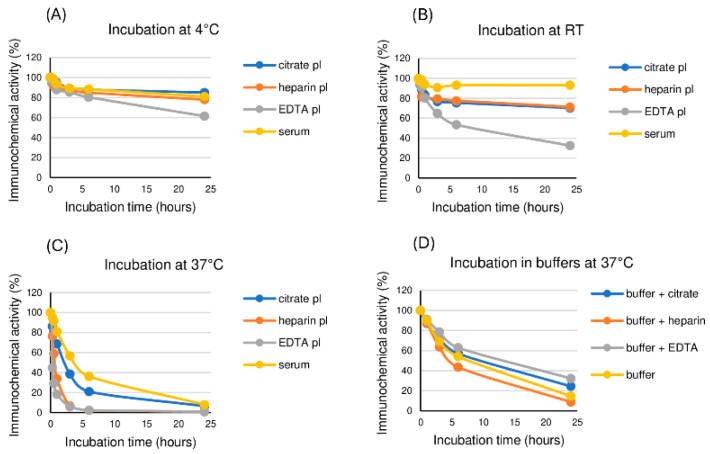
Time course of immunochemical activity of the ITC-complex upon incubation in different blood samples and in buffers with anticoagulants. The ITC-complex at a concentration of 3 ng/mL was incubated in citrate, heparin, or EDTA plasmas, and in serum at 4 °C (**A**), RT (**B**), and 37 °C (**C**) or in buffers with anticoagulants at 37 °C (**D**). Immunoreactivity in the samples was measured by CLIA using the Tcom8-TnT7E7 assay.

To study the stability of the ITC-complex in blood samples of various types, the ITC-complex was spiked into normal serum or normal plasmas containing citrate, heparin, or EDTA to a final concentration of 3 ng/mL and incubated for up to 24 h at 4 °C, room temperature (RT), and 37 °C. As controls, the ITC-complexes were spiked into buffer solutions containing corresponding concentrations of sodium citrate, heparin, or EDTA. Immunochemical activity was measured in aliquots collected after 0.25, 0.5, 1, 3, 6, and 24 h of incubation using the Tcom8-TnT7E7 assay, which is specific for the ternary ITC-complex. The activity of the ITC-complex did not change considerably after incubation at +4 °C and RT in serum or citate and heparin plasmas but decreased at +37 °C with a T½ of 4.0, 2.5, and 1.0 h, respectively. The activity of the ITC-complex in EDTA plasma decreased at all tested temperatures with a T½ of more than 24 h at 4 °C, 6.0 h at RT, and 0.3 h at 37 °C (Figure 1). The activity of the ITC-complex also decreased at 37 °C in the buffers with or without anticoagulants, but the decrease was much slower than in the corresponding plasmas: 9.2, 4.7, 13.3, and 7.9 h for buffers with sodium citrate, heparin, EDTA, or buffer alone, respectively. The decrease in ITC-complex activity at 37 °C could be explained either by the dissociation of the ITC-complex or by the degradation of the proteins it contains. Moreover, some plasma components appeared to promote the dissociation/degradation of the ITC-complex.

### 2.2. Investigation of Troponin Degradation via Western Blotting

To investigate if cTnI or cTnT degrade in blood samples, troponins immunoprecipitated from the samples after in vitro incubation at 37 °C were examined using SDS-PAGE followed by western blotting.

**Figure 2:**
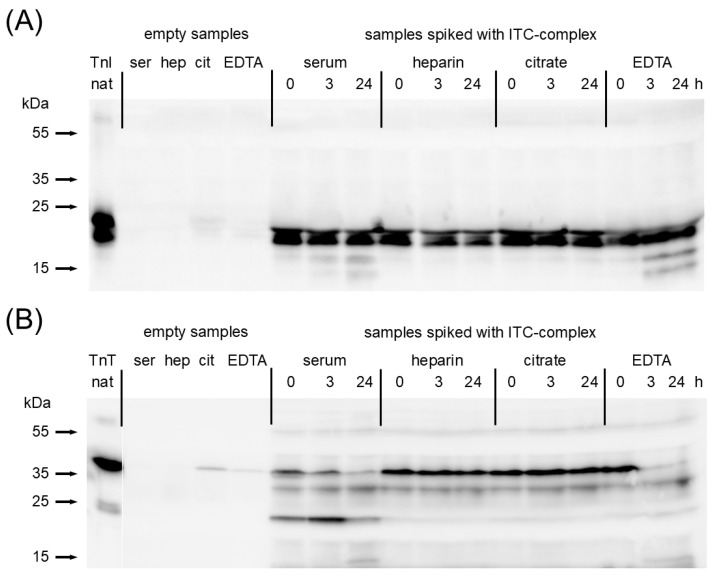
Western blotting analysis of cTnI and cTnT degradation upon incubation in different matrices. (**A**) cTnI was stained with TnI560 mAb: (**B**) cTnT was stained with TnT329 and TnT7E7 mAbs.

No degradation of cTnI was found in citrate or heparin plasmas after incubation for 24 h, whereas partial proteolysis was observed in the serum and EDTA plasma (Figure 2). Degradation of cTnT also did not occur in citrate and heparin plasmas but most cTnT was proteolyzed in the serum and EDTA plasma, even after 3 h of incubation. These results showed a decrease in ITC-complex activity in citrate and heparin plasmas which could not be explained by the degradation of cTnI or cTnT; therefore, the dissociation of proteins from the complex is likely the major driving factor. However, both dissociation and degradation of the ITC-complex may occur in the serum or EDTA plasma. 

### 2.3. Investigation of ITC-Complex Dissociation via Gel Filtration (GF)

**Figure 3:**
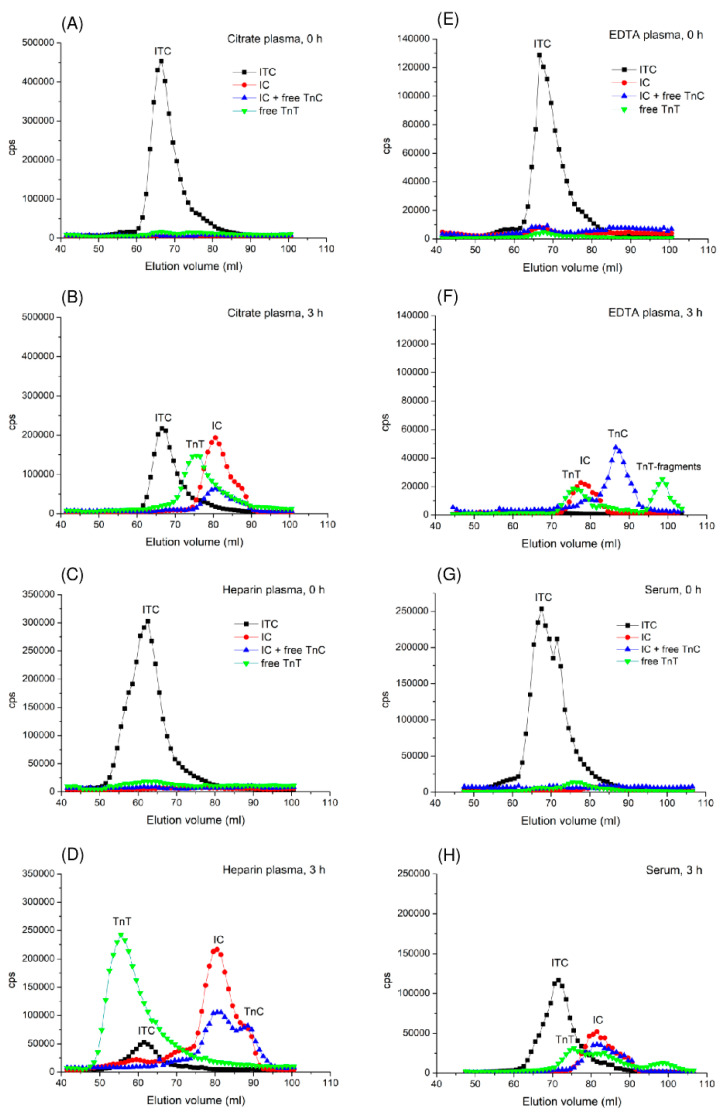
Detection of the ITC-complex dissociation products in the GF profiles before and after 3 h of incubation at 37 °C in different matrices. The ITC-complex spiked in citrate plasma (**A**,**B**), heparin plasma (**C**,**D**), EDTA plasma (**E**,**F**) and serum (**G**,**H**), and troponin immunoreactivity in the GF fractions was measured via FIA using the Tcom8-TnT7E7 (specific to the ITC-complex, black), TnI84-TnC7B9 (specific to the IC-complex, red), TnC12G3-TnC7B9 (specific to the IC-complex and free TnC, blue), and TnT155-TnT7E7 (specific to free cTnT, green) assays.

To study the changes in troponin composition upon incubation at 37 °C, we used GF to analyse the products of ITC-complex degradation/dissociation. One microgram of the ITC-complex was spiked into 1 mL of normal citrate, heparin, or EDTA plasmas or serum and subjected to HPLC GF before and after 3 h of incubation at 37 °C. The resulting profiles were analysed for the ITC-complex (Tcom8-TnT7E7 assay), IC-complex (TnI84-TnC7B9 assay), and free cTnT (TnT155-TnT7E7 assay) and TnC (TnC12G3-TnC7B9 assay). The immunoreactivity profiles of the samples gel-filtered before incubation at 37 °C contained only the peak of the ternary ITC-complex, whereas no peaks corresponding to the binary IC-complex or free cTnT were observed. In citrate-treated plasma, after 3 h of incubation at 37 °C, the appearance of the IC-complex and free cTnT confirmed the hypothesis regarding the dissociation of cTnT from the ITC-complex although a large amount of the ITC-complex remained present in the sample. In heparin-treated plasma, only a small amount of the ITC-complex remained after incubation, and the primary detected proteins were the IC-complex and free cTnT. In EDTA plasma, the ITC-complex completely transformed into the IC-complex and free cTnT, and some part of the cTnT was cleaved into several fragments (Figure 3). It should be noted that in the presence of heparin, the ITC-complex and free cTnT eluted in smaller elution volumes, most likely owing to heparin binding to cTnT. Incubation of the ITC-complex in serum also resulted in the formation of the IC-complex and free cTnT and its fragments, but the N-terminal part of cTnT in the remaining ITC-complex was cleaved by thrombin [17], with a shift in the elution volume of the peak corresponding to the ITC complex from ~68 to ~72 mL, and the appearance of some fragments of cTnT.

These data show that the ITC-complex dissociates in serum and plasma upon incubation at 37 °C to form a binary IC-complex and free cTnT, although cTnT also degrades in serum and EDTA plasma. Moreover, the IC-complex undergoes further dissociation in heparin-treated and especially, in EDTA plasma. At the same time, we did not detect dissociation of the IC-complex in the citrate-treated plasma or serum.

### 2.4. Dissociation of the Endogenous ITC-Complex in Citrate and Heparin Plasma of Patients with AMI

**Figure 4:**
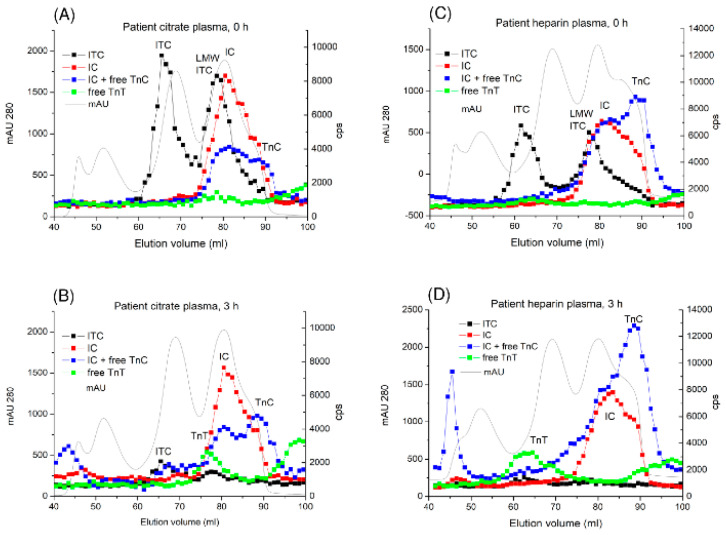
Detection of endogenous troponin complex dissociation products from GF. Citrate plasma sample (**A**,**B**) and heparin plasma (**C**,**D**) from a patient with AMI were incubated at 37 °C, and troponin immunoreactivity in the GF fractions was measured by FIA using the Tcom8-TnT7E7 (specific to the ITC-complex, black), TnI84-TnC7B9 (specific to the IC-complex, red), TnC12G3-TnC7B9 (specific to the IC-complex and free TnC, blue) and TnT155-TnT7E7 (specific to free cTnT, green) assays. Solid grey line–protein profile (mAU280).

To confirm whether the endogenous ITC-complex in plasma from patients behaves in the same manner as the isolated complex spiked into normal citrate and heparin plasma, samples taken from patients with AMI were subjected to GF before and after 3 h of incubation at 37 °C. In the initial samples, full-size and LMW ITC-complexes, IC-complex, and free TnC were detected. Free cTnT was not detected in any of the samples. After 3 h of incubation at 37 °C, very little amounts of full-size and LMW ITC-complexes remained in the citrate plasma samples and almost no complex remained in the heparin-treated plasma samples. The amount of the IC-complex and free TnC increased, and free cTnT and its fragments appeared. After incubation, some high molecular weight complexes containing TnC were detected (peaks at 43–45 mL), which may be explained by complex formation between TnC or the IC-complex and some high-molecular-weight plasma proteins (representative profiles are shown in Figure 4). Our results indicate that the endogenous ITC-complex dissociates in blood samples at 37 °C similar to the ITC-complex which spiked in normal plasmas. 

### 2.5. Influence of Anti-Troponin Autoantibodies on ITC-Complex Dissociation

**Figure 5:**
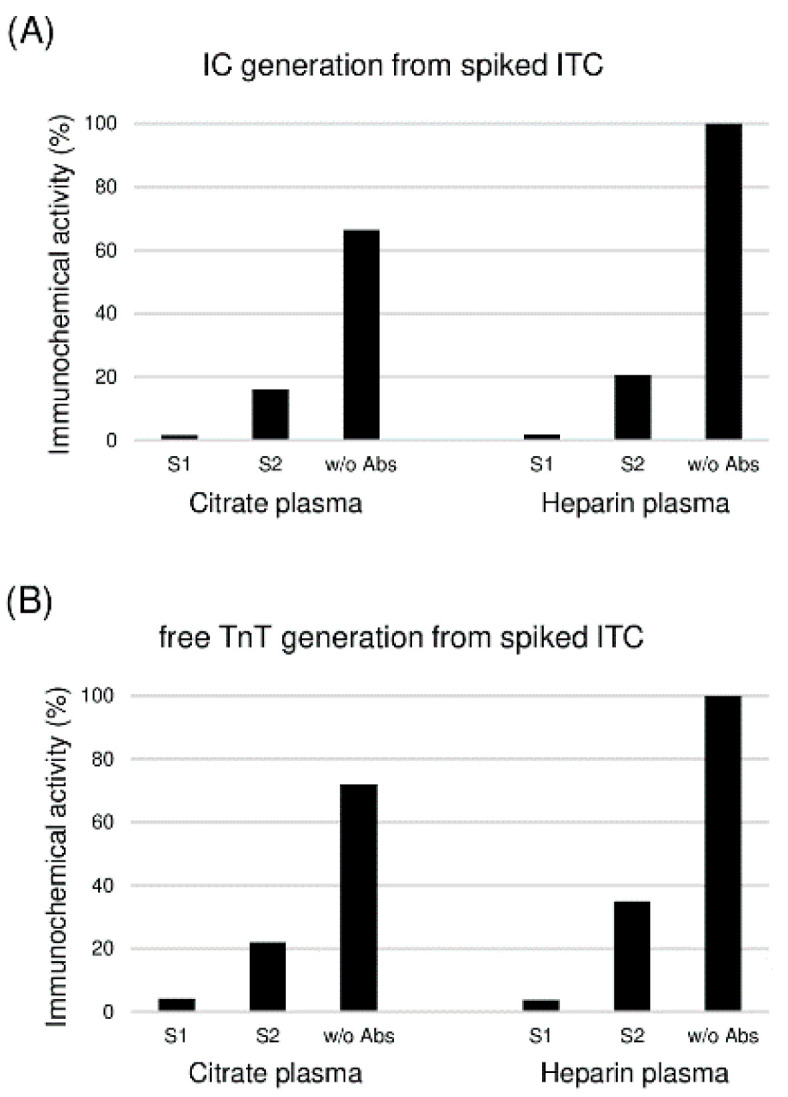
Influence of autoantibodies on ITC-complex dissociation. The generation of the IC-complex (**A**) and free cTnT (**B**) after 3 h of incubation of the ITC-complex in citrate and heparin plasma samples containing autoantibodies (S1 and S2) were detected via FIA using the TnI84-TnC7B9 and TnT155-TnT7E7 assays, respectively. Signals in normal heparin plasma samples were taken as 100%.

As some blood samples may contain autoantibodies that bind to the ITC-complex via its common cTnI-cTnT epitope [18], we investigated the possible influence of these antibodies on the dissociation of the ITC-complex. For this purpose, the ITC-complex was spiked into citrate and heparin plasma samples taken from two healthy individuals with anti-troponin autoantibodies in their blood. In contrast to the control pooled plasma sample taken from healthy donors not having autoantibodies, almost no generation of the IC-complex or free cTnT was observed after a 3 h incubation period of the samples at 37 °C (Figure 5).

### 2.6. Identification of Components That May Promote ITC-Complex Dissociation in Plasma Samples

**Figure 6:**
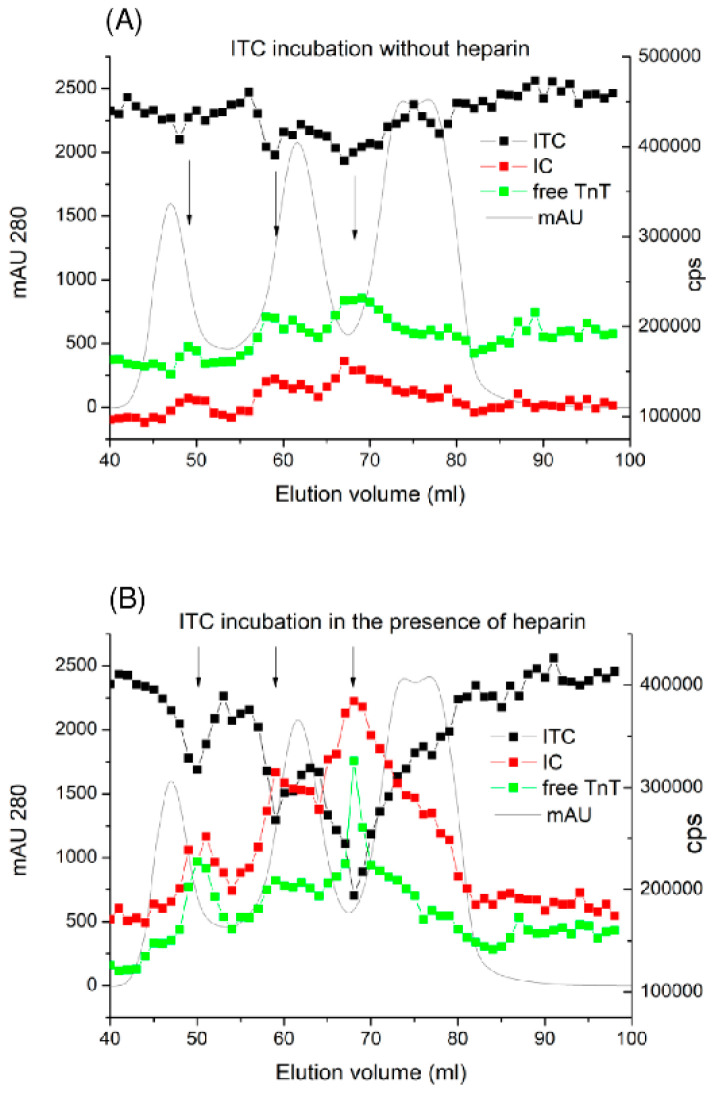
Identification of plasma fractions that promote ITC-complex dissociation. The ITC-complex was added to all GF fractions and incubated for 3 h at 37 °C in the absence (**A**) or presence (**B**) of heparin. Troponin immunochemical activity was measured via FIA using the Tcom8-TnT7E7 (specific to ternary ITC, black) TnI84-TnC7B9 (IC-complex, red), and TnT155-TnT7E7 (free cTnT, green) assays. Grey line–protein profile (mAU 280). Fractions that promote ITC-complex dissociation are indicated by arrows.

To determine which of the plasma fractions could promote the dissociation of cTnT from the ITC-complex, GF of a normal citrate plasma sample was performed. Equal volumes of 150 ng/mL of the ITC-complex in buffer solution with or without heparin were added to all fractions. After 3 h of incubation at 37 °C, the ITC- and IC-complexes and free cTnT were measured in all fractions. We observed that several plasma fractions enhanced ITC-complex dissociation (Figure 6). Dissociation of these fractions was highly pronounced when the ITC-complex was in the presence of heparin. However, we could not identify the specific plasma factors that caused ITC dissociation in these fractions, and further studies are needed to clarify these components.

## 3. Discussion

Both ternary and binary troponin complexes were previously found in the blood samples of patients with AMI [16], but it was not clear if their ratio in the samples corresponded to their ratio in circulation. Whether the sample preparation or storage method can cause the transition of one troponin complex to another or lead to the degradation of troponin molecules is also of interest. Previously, we showed that cTnT is cleaved by thrombin during serum sample preparation and that its forms in the test samples do not correspond exactly to those present in the bloodstream [17]. However, the influence of the different anticoagulants used for plasma preparation on the integrity of troponin complexes has not yet been investigated.

Our current study revealed that the incubation of the ITC-complex in buffers with or without anticoagulants at 37 °C led to a decrease in ITC-complex activity. This decline was highly pronounced in heparin and EDTA plasmas. Observed decrease could not be explained by troponin degradation, at least in the citrate- and heparin-treated plasma, because no degradation of cTnI or cTnT was detected by western blotting after the 24-h incubation of these proteins in the corresponding plasmas. Some degradation of cTnI or cTnT took place in EDTA plasma and serum; however, it occurred much later than the decrease in ITC complex activity. A study on the time-course of cTnT degradation by EDTA was recently been published [19] where it was shown that cTnT proteolysis becomes significant only after several hours of incubation. This, together with the data obtained in the present study, allows us to presume that the dissociation of cTnT from the ITC-complex precedes its degradation (at least in most of the sites of proteolysis). In contrast, in serum, partial degradation of cTnT occurs almost instantaneously due to the thrombin activity.

Gel filtration analysis of the ITC-complex incubated in plasma prepared using different anticoagulants and in serum showed that the activity of the ITC-complex decreased, together with an increase in the activities of the binary IC-complex and free cTnT. Thus, we concluded that cTnT dissociates from the ITC-complex without degradation, at least in citrate and heparin plasmas. However, cTnT dissociation in EDTA plasma and serum was accompanied by its proteolysis. After incubation of the ITC-complex in serum and citrate plasmas for 3 h at 37 °C, we only observed the appearance of the IC-complex and free full-sized cTnT or its fragments, whereas incubation of the ITC-complex in EDTA or heparin plasmas led to the further dissociation of the IC-complex with the formation of free TnC. The dissociation of the ITC-complex was also observed for the endogenous ITC-complex when citrate and heparin plasma samples from patients with AMI were incubated for 3 h at 37 °C. Both forms of the ternary ITC-complex (full-size and LMW ITC-complexes) disappeared, and the amounts of binary IC-complex and free cTnT increased. Some portion of the IC-complex apparently also dissociated to give rise to free TnC, not only in heparin-treated plasma samples but also in citrate-treated plasma samples (even before in vitro incubation). We speculate that citrate-treated plasma samples collected from patients shortly after AMI onset contained heparin administered according to the treatment protocol.

The dissociation of the ITC-complex in EDTA plasma was not surprising because calcium ions are crucial for ITC-complex integrity, and disruption of the ITC-complex by EDTA into individual subunits has been previously reported [20]. However, the strong enhancement of ITC-complex dissociation in the presence of heparin was unexpected. As a significant difference was absent in the dissociation of the ITC-complex in buffers with or without heparin, we suggest that some plasma components could be involved in this process. An attempt to identify these components revealed that at least three fractions in the blood samples enhanced the dissociation of the ITC-complex. In all fractions, ITC-complex dissociation was highly pronounced in the presence of heparin. These data may explain the previous finding that free cTnT can be extracted from cardiac tissue using a large volume of serum warmed up to 37 °C [21].

The diagnostic value of different troponin complexes and the differential detection of free troponins has been discussed previously [22,23]. However, one can suggest that ITC-complex dissociation may continue in blood samples obtained from patients and distort the results of the ITC-complex analysis, leading to misinterpretations. However, our experiments showed that dissociation of the ITC-complex in citrate and heparin plasmas and in serum occurs only at 37 °C and is negligible at 4 °C or at room temperature even after 24 hours of incubation. It is also worth noting that we observed the dissociation of the ITC- and IC-complexes after a rather long incubation period (3 h), and we presume that this dissociation would not be pronounced after a short (5–10 min) incubation during the modern immunochemical assay procedure, especially in the presence of heparin-blocking agents.

Dissociation of cTnT from the ITC-complex is greatly impeded during incubation in the presence of anti-troponin autoantibodies which may be present in the blood of patients with AMI and healthy individuals. This may be explained by the fact that anti-troponin autoantibodies interact with a spatial epitope that includes parts of the cTnI, cTnT, and possibly TnC molecules [18], thereby connecting troponins and preventing their dissociation.

In our previous work, we showed that ternary full-size and LMW ITC-complexes are the predominant troponin forms at the early stage of AMI, whereas at later stages, the binary IC-complex becomes the main detectable form [16]. However, whether the different troponin forms released from damaged myocardium at different stages of AMI or the ternary ITC-complex could be converted into the binary IC-complex in the bloodstream upon disease progression was unclear. We suggest that dissociation of the ITC-complex may, to some extent, occur in the circulation, especially if heparin is administered to patients with AMI at the beginning of AMI treatment. This hypothesis is confirmed by our results, where the endogenous ITC-complex in blood samples taken from patients with AMI dissociated similarly to the native ITC-complex spiked into normal blood plasma samples. However, this does not contradict the suggestion that the IC-complex can also be released from the myocardium at later stages of AMI. It is most likely that both processes occur in patients with AMI.

## 4. Materials and Methods

### 4.1. Reagents

Unless otherwise stated, all chemicals were obtained from Sigma-Aldrich (St. Louis, MO, USA). The native cardiac troponin complex was obtained from HyTest (Turku, Finland). Buffer A (20 mM Tris-HCl, pH 7.5, containing 150 mM KCl, 5 mM CaCl_2_, 75 g/L bovine serum albumin (BSA), and 0.15 g/L NaN_3_) was used for dilution. To prepare buffer solutions containing sodium citrate, heparin, or EDTA, 9 mL of Buffer A was added to corresponding 9-mL Vacuette tubes (Greiner Bio-One, Kremsmünster, Austria) and incubated for 15 min at room temperature with shaking.

Anti-TnI monoclonal antibodies (mAbs): TnI84 (specific to amino acid residues (aar) 117–126); anti-TnT mAbs: TnT329 (aar 119–138), TnT7E7 (aar 223–242), TnT155 (aar 263–281), anti-TnC mAbs TnC12G3, TnC7B9, and mAb Tcom8 specific to the cardiac IC- and ITCcomplexes were obtainedfrom HyTest (Turku, Finland). 

### 4.2. Blood Samples

Normal pooled citrate, heparin and EDTA plasma, and serum samples were prepared from the blood of 15 healthy volunteers using a standard technique with corresponding vacutainers (Greiner Bio-One, Kremsmünster, Austria). Heparin plasma samples containing high concentrations of anti-troponin complex autoantibodies were obtained from two healthy individuals. Citrate- and heparin-treated plasma samples from patients with AMI were collected 3–7 h after AMI onset after revascularisation of the coronary arteries, according to the Protocol#292 from 25 September 2023, approved by the Independent Ethical Committee of the National Medical Research Centre of Cardiology named after academician E.I. Chazov, Moscow, Russia. All participants were informed about the study in accordance with the current revision of the Helsinki Declaration [24].

### 4.3. Sandwich Fluoroimmunoassay (FIA)

A two-step sandwich FIA was performed. Capture antibodies at a concentration of 10 μg/mL in phosphate-buffered saline (100 μL per well) were added to the wells of 96-well polystyrene plates (Corning Costar, New York, NY, USA) and incubated for 30 min at room temperature (RT) under gentle shaking. The plates were washed thrice with a washing solution (50 mM Tris-HCl buffer, pH 7.8, containing 150 mM NaCl and 0.25 g/L Tween 20). Following washing, 50 μL of the sample and 50 μL of detection antibodies (200 ng/well) labelled with a stable Eu^3+^ chelate in the assay buffer (50 mM Tris-HCl buffer, pH 7.5, containing 150 mM NaCl, 0.01% Tween 40, 5 g/L BSA, and 0.5 g/L NaN_3_) were added to the wells and incubated for 1 h at RT under shaking. Following six washes with the washing solution, 200 μL of an enhancement solution (50 mM glycine-NaOH buffer, pH 10.0, containing 5 mM Na_2_CO_3_, 50 ml/L glycerol, 200 ml/L 1-propanol, 1 M NaCl, and 1.75 M NaSCN) was added per well and incubated for 3 min under intensive shaking at RT. Fluorescence was measured using a 1420 Multilabel Counter Victor instrument (PerkinElmer, Waltham, MA, USA).

### 4.4. Chemiluminescence Immunoassay (CLIA)

Microtitre plates with 96 wells were coated with monoclonal antibody Tcom8 (specific to the conformational epitope formed by cTnI and TnC in binary IC and ternary ITC complexes) as a capture antibody (1 μg/mL in phosphate-buffered saline, 50 μL per well) for 1 h. After washing with phosphate-buffered saline containing Tween 20 (PBST), 25 μL of antigen solution and 25 μL of biotinylated anti-cTnT mAb TnT7E7 (2 μg/mL in PBST containing 75 g/L BSA) were applied and incubated for 45 min. After washing with PBST, 50 μL of streptavidin poly-HRP solution diluted to 1:100,000 in PBST containing 7.5% BSA was added to each well. After incubation for 10 min and washing, SuperSignal™ ELISA Femto Substrate (Thermo Fisher Scientific, Waltham, MA, USA, 50 μL per well, 1-min incubation) was used for immune complex detection. The luminescence in each well was measured using a Victor 1420 multilabel counter (PerkinElmer, Waltham, MA, USA).

The Tcom8-TnT7E7 assay is specific to the ternary troponin complex, in which cTnI, cTnT, and TnC bind together. The TnI84-TnC7B9 assay detects the binary IC-complex. The TnT155-TnT7E7 assay detects only free cTnT and not the ITC-complex. The TnC12G3-TnC7B9 assay detects free TnC and TnC in the binary IC-complex.

The half-life (T½) of the ITC-complex was calculated as the decrease in immunological activity using the initial linear parts of the graphs that were described by the equation for first-order rate reactions.

### 4.5. Gel Filtration (GF)

Gel filtration was performed using an AKTA Pure Chromatography System (GE Healthcare) on a HiLoad Superdex 200 PG 16/60 column (GE Healthcare, Chicago, IL, USA). Samples (0.1–1.0 mL) were loaded on the column that was equilibrated with 50 mM Tris-HCl buffer, pH 7.5, containing 150 mM NaCl, 5 mM CaCl_2_, 1 g/L BSA, and 0.15 g/L NaN_3_. Proteins were eluted at a rate of 2 mL/min, and 1 mL fractions were collected and analysed using the FIA.

### 4.6. Immunoextraction of the ITC-Complex and Its Analysis in Western Blotting

Immunoextraction of the ITC-complex was performed using an immunoaffinity sorbent containing anti-cTnI and anti-cTnT mAbs, as described previously [14,15]. In brief, extracted proteins were separated using 10–20% Tris-Glycine SDS-PAGE, blotted onto a 0.45 μm nitrocellulose membrane (Bio-Rad, Hercules, CA, USA) and stained with biotinylated anti-cTnI (TnI560) or anti-cTnT (TnT329 and TnT7E7) mAbs. After incubation with streptavidin poly-HRP Thermo Fisher Scientific (Waltham, MA, USA), protein bands were visualised using SuperSignal West Femto Maximum Sensitivity Substrate (Thermo Fisher Scientific, Waltham, MA, USA) on a ChemiDoc Touch Imaging System (Bio-Rad, Hercules, CA, USA).

## 5. Conclusions

(1) The ITC-complex spontaneously dissociates at 37 °C in the buffer, citrate, heparin, and EDTA plasmas, and in serum to form the IC-complex and free cTnT; (2) the IC-complex further dissociates in heparin and EDTA plasmas at 37 °C to form free TnC; (3) blood plasma contains at least three protein fractions that enhance the dissociation of the ITC-complex into free TnT and the IC-complex, especially in the presence of heparin; (4) the prevalence of the IC-complex in blood of patients with AMI at later stages of the disease can occur owing to both its release from the damaged myocardium and dissociation of the ITC-complex in the circulation; (5) ITC-complex is mostly stable in vitro at room temperature or at 4 °C in the citrate- and heparin-treated plasma and serum samples in the first 24 h of incubation; (6) anti-troponin autoantibodies impede the dissociation of cTnT from the ITC-complex and seem to increase its stability in the blood stream.

## Data Availability

The raw data supporting the conclusions of this article will be made available by the authors on request.
